# Modeling Thermodynamic Behavior of Ultrathin Films: Comparison with Experiments

**DOI:** 10.1002/cphc.202500199

**Published:** 2025-07-16

**Authors:** Modibo Camara, Elian Masnada, Sophie Cantin, Odile Fichet

**Affiliations:** ^1^ CY Cergy Paris Université LPPI F95000 Cergy France

**Keywords:** pressure–, area isotherms, thermodynamic models, thicknesses

## Abstract

This work aims to model the thermodynamic behavior of ultrathin polymer films. A mean‐field thermodynamic model, originally developed for 3D polymers and parametrized by fitting the 3D pressure–volume–temperature data, is adapted to the 2D case. Remarkably, without any adjustable parameters, the 2D model successfully describes the experimental pressure–area isotherms for a wide range of polymers (polydimethylsiloxane (PDMS), polymethylmethacrylate (PMMA), polytetrahydrofuran, and polybutadiene) with varying hydrophobic–hydrophilic balances. Furthermore, it allows for the prediction of ultrathin film thickness, yielding values consistent with available experimental data for different polymers (PDMS, PMMA, and polybutadiene, especially). This 2D model remains applicable across different phases of the phase diagram, provided the film is in a dense state.

## Graphical abstract

A 3D thermodynamic model in adapted to 2D ultrathin polymer films. Compression isotherms of various polymers are fitted without adjustable parameters. The model predicts polymer film thickness agrees with experiments.

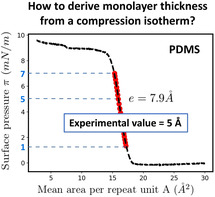

## 1 Introduction

Ultrathin films at the air–water interface provide a versatile platform for investigating surface phenomena.^[^ ^1^, ^2^, ^3^ ^]^ Their well‐defined structure and customizable properties make them essential in a variety of applications, from biosensors and drug delivery systems to advanced thin film coatings.^[^ ^4^, ^5^ ^]^ These films allow for a precise control over surface characteristics like surface tension and reactivity, making them critical for understanding interfacial processes. Their simplicity also accelerates experimentation and analysis, streamlining the development of innovative materials and technologies.^[^ ^6^ ^]^ In summary, these ultrathin films at the air–water interface are pivotal in advancing interfacial science and hold significant promise across multiple industries, offering a range of practical applications.

To characterize such ultrathin films made of water‐insoluble molecules, the surface pressure–area isotherm, which monitors the change in surface pressure as the area available for the molecules at the interface is reduced, provides information on the film density and compressibility in each 2D phase. Several in situ techniques are used to characterize the molecular organization and film morphology at different length scales. For example, Brewster angle microscopy allows visualization of the phases and phase transitions at the mesoscopic scale.^[^ ^7^, ^8^ ^]^ Based on the intensity of the light reflected by the interface, it is sensitive to the thickness and the density of the film but remains nonquantitative. Polarization‐modulated infrared reflection–absorption spectroscopy and sum frequency generation provide information about the molecular‐scale arrangement by probing the orientation of the chemical bonds.^[^ ^9^, ^10^ ^]^ However, determining the thickness of such ultrathin films is much more complicated. Various techniques that exploit different principles and methodologies are available.^[^ ^6^, ^11^ ^]^ For instance, ellipsometry measures changes in polarized light after interaction with the film.^[^ ^12^ ^]^ However, determining the thickness is done with a model that requires knowledge of the refractive index of the system.^[^ ^13^ ^]^ X‐ray and neutron reflectivity techniques also offer high‐resolution insights into the vertical structure.^[^ ^11^ ^]^ They can be conducted directly at the air–water interface but only on large‐scale facilities. In addition, for in situ characterizations at the air–water interface, reliable measurements on such ultrathin layers require a sufficient contrast of the film with both the air and the water. Consequently, X‐ray reflectivity is better suitable for molecules with a high electronic density, which is not the case of most studied polymers, while deuterated molecules have to be used for neutron reflectivity. The analysis of the reflectivity curves is also not straightforward, and thickness determination requires modeling using several physical parameters such as electronic density or scattering length density and roughness at the different interfaces. The thickness of the thin film at the air–water interface can also be measured through imaging techniques after transfer of the film onto a solid substrate. For example, atomic force microscopy and scanning tunneling microscopy provide topographical information with a nanoscale vertical resolution.6 Due to the change of substrate, i.e. solid instead of water, and possible molecular reorganization during transfer, the obtained thickness should be carefully transposed to the film thickness in situ at the air–water interface. Thus, in situ thickness measurement of such nanometer‐thick layers is not straightforward, requiring knowledge or modeling of some layer parameters, and in some cases, complex experimental set‐up and/or time‐consuming analysis.

As mentioned previously, these ultrathin films have been widely used as a model, and they were also modeled.^[^ ^14^, ^15^ ^]^ Several theoretical models were proposed to describe the main phase transitions.^[^ ^16^, ^17^, ^18^ ^]^ However, the implementation of thermodynamic theory for the description of real monolayers is hampered by the fact that the resulting equations are extremely cumbersome with several unknown parameters. Indeed, some of these models seek to explicitly consider the interface between the film and water, either by molecular dynamics or in a coarse‐grained manner to obtain the detailed structure of the films.^[^ ^19^, ^20^, ^21^ ^]^ However, none of the proposed models allows assessing the thickness of the ultrathin film.

Furthermore, while the organization with respect to the water surface of usual amphiphilic molecules, such as fatty acids or phospholipids, can be predicted easily from their chemical structure and the surface density determined from thermodynamic measurements, that of polymers is more complicated to assess.^[^ ^22^ ^]^ Indeed, the hydrophilic and hydrophobic parts are often less well‐defined, or alternated one after the other, making the organization more random and difficult to predict.

Thus, we sought to thermodynamically model an ultrathin polymer film at the air–water interface, allowing to determine its thickness simply from its compression isotherm. To achieve this, a mean field semiquantitative thermodynamic model, originally developed for 3D compressible and amorphous polymers in a fluid state above the glass transition temperature, was employed.^[^ ^23^ ^]^ In 3D, this thermodynamic model reproduces pressure–volume–temperature (PVT) data, i.e. the evolution of pressure as a function of volume at fixed temperature, for a given polymer described by the van der Waals interaction energy *a* between repeat units and the close packing parameter $\rho_{0}$ (density of repeat units at zero free volume, i.e. infinite pressure). The model has been applied to polymer monolayers at the air–water interface, replacing pressure *P* by surface pressure π and volume *V* by mean area per repeat unit *A*. The *a* and $\rho_{0}$ parameters were thus assumed to be similar. Thus, from the *a* and *ρ*_*0*_ values, π=f(A) isotherm for polymer monolayer was calculated in a dense phase, with only the thickness of the polymer layer *e* as an adjustable parameter. Various polymers were examined, and the theoretical thickness of the thin films was ultimately compared to experimental measurements found in the literature.

## 2 Results and Discussion

We aimed to develop a thermodynamic model describing ultrathin polymer films at the air–water interface. As monolayer‐thick films, they form 2D model systems, and the theory developed here is notably used to estimate their thickness directly from the compression isotherm. One should mention that polymer–polymer interactions in the monolayer will be described by the *a* parameter, while polymer/water interactions will be considered in the application of the model to experimental π–A isotherms. Indeed, these interfacial interactions play a significant role in the shape of these isotherms, i.e., the area per repeat unit *A* at which the surface pressure starts to rise and the slope of curve when the monolyer is in a dense state. In addition, the *A* value measured just before the polymer monolayer collapse, i.e., 2D to 3D transition, is also linked to the close packing parameter *ρ*_*0*_. So even though not all interactions are explicitly mentioned in the model, they are implicitly considered in its application to experimental measurement of π=f(A) isotherms.

Our approach here is to describe such ultrathin polymer films using a thermodynamic model published in 2014 by Masnada et al. and initially developed for polymer blends.^[^ ^23^ ^]^ This model was chosen because it allows modeling pure compressible polymers in a simple way with no adjustable parameters, since the model's parameters are determined by fitting the 3D PVT data. Thus, the close‐packing parameter $\rho_{0}$ corresponding to the density of repeat units at zero free volume, i.e. infinite pressure, and the *a* parameter describing the interactions between repeat units were determined using the PVT data of the polymer computed through Tait's equation. Then, these variables were translated as the parameters to describe the experimental compression isotherm of the film.

### 2.1 Overview of the Properties of the Four Polymers Studied in Ultrathin Film at the Air–Water Interface in Relation to the Model

**Figure 1:**
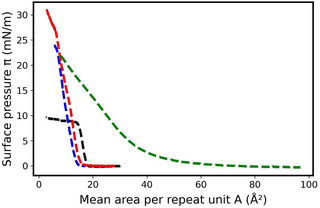
Experimental surface pressure π–mean area per repeat unit A isotherms for PDMS (black line), atactic PMMA (red line), PTHF (green line), and 1,2‐PB (blue line) monolayers at 20 °C.

**Figure** **1** shows the surface pressure π–mean area per repeat unit A curves for polydimethylsiloxane (PDMS), atactic polymethylmethacrylate (PMMA), polytetrahydrofuran (PTHF), and 1,2‐PB measured at the air–water interface at 20 °C.

At the highest considered *A* values and close to zero surface pressure, a dilute gas state and a dense state coexist. Then, the dense state progressively occupies a higher surface fraction of the film as *A* decreases, in the same way as for a gas–liquid first‐order phase transition in 3D. Upon further compression, the surface pressure starts rising at *A* values dependent on the polymer and marking the beginning of the formation of a uniform dense state or so‐called semidilute regime.^[^ ^22^ ^]^ In this regime, the polymer chains are in contact with each other and progressively interpenetrate as the surface pressure increases. It can be noticed that the area per repeat unit *A* at which the semidilute regime starts and the slope of the curve in this regime are strongly dependent on the polymer.

In the literature, it has been reported that depending on whether the water behaves as a poor, good, or theta solvent to the polymer, the slope of the π=f(A) isotherm is different.^[^ ^22^ ^]^ The higher the hydrophobicity of the polymer, the less the water is a good solvent. As a result, even if the conformation between polymer and fatty acids is of course very different, more hydrophilic polymer films behave as the so‐called liquid expanded phase of fatty acids monolayers, whereas more hydrophobic polymer films have a liquid condensed phase behavior. For example, the isotherm slope of polytertbutylacrylate (PTBA) under good solvent conditions is lower than that of more hydrophobic polytertbutylmethacrylate (PTBMA). Indeed, the PTBA chains are swollen and spread on the surface, while unfavorable interactions with water make the PTBMA chains occupy a smaller area.^[^ ^24^ ^]^ Thus, the interactions between the polymer and water can be directly related to the isothermal compressibility of the polymer monolayers defined as

(1) $$C_{S}=-\frac{1}{A}\times \frac{\partial A}{\partial \pi }{|}_{T}$$

The values of $C_{S}$ of the four polymer monolayers are deduced from the isotherms and reported in **Table** **1**, as well as the studied ranges of surface pressure. These $C_{S}$ values are clearly decreasing as PTHF > PMMA > 1,2‐PB > PDMS, i.e., with the increasing hydrophobic character of the polymer. This highlights a large range of behavior in the semidilute regime for these four polymer monolayers, which makes them interesting to model thermodynamically.

**Table 1:** Compressibility $C_{S}$ at 20 °C for the different polymer monolayers in the semidilute regime.

Polymer	Range of surface pressures [mN m^−1^]	$C_{S}$ [m N^−1^]
PDMS	1–7	20
PB	3–20	25
PMMA	3–9 and 20–25	25 and 40
PTHF	5–20	55

Finally, above a certain surface pressure, depending on the polymer, an inflection, or a plateau for the PDMS, is observed, which indicates the collapse of the monolayer, also described as the concentrated regime.

The aim of this article is to model the semidilute regime of the monolayer in which the surface pressure increases continuously. It should be emphasized that the four polymer monolayers studied are in this regime, as the measurement temperature (20 °C) is above their glass transition temperature.^[^ ^22^ ^]^ This is indeed the case for PDMS, PTHF, and 1,2‐PB, whose bulk glass transition temperatures (Tg) are −127, −80, and −102 °C, respectively. For atactic PMMA, the Tg, which ranges between 104 and 108 °C in bulk, has been reported, as for several polymers, to be significantly lower in monolayer, around 11 °C.^[^ ^22^ ^]^ Consequently, all the polymers are in a fluid state in the monolayer at the air–water interface at 20 °C.

### 2.2 Transition from 3D Isotherms to 2D Ultrathin Films—Methodology and Application to PDMS

Our approach first aims to determine the theoretical thickness of a monolayer of PDMS at the air–water interface. Despite its low surface free energy due to its predominantly hydrophobic character, the PDMS polymer forms a stable monolayer, and the determination of its organization at the air–water interface has been the subject of numerous experimental studies.^[^ ^25^, ^26^, ^27^, ^28^ ^]^ Indeed, its poorly defined hydrophilic and hydrophobic regions pose challenges in accurately predicting its organization at the air–water interface and understanding its interfacial behavior.

#### 2.2.1 Determination of the Parameters a and $\rho_{0}$ from 3D Thermodynamic

**Figure 2:**
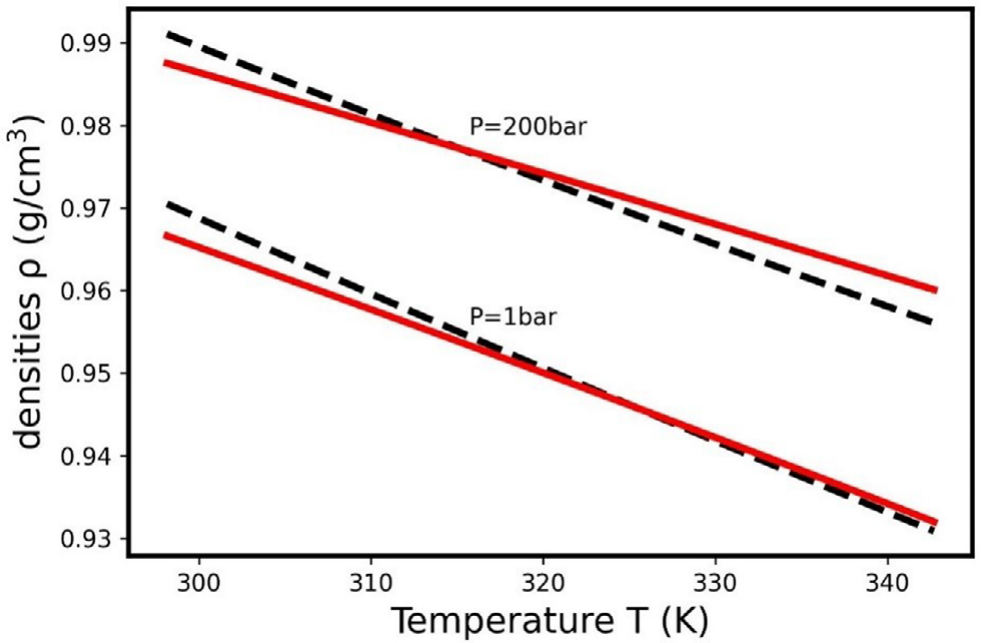
Comparison between the experimental density of the PDMS for $X_{A}=184$ (dashed black curves) and its best fit (solid red curves) at 1 and 200 bar with $a=-1.557\times 10^{-20}J$ and $\rho_{0}=9.310\times 10^{27}m^{-3}$.

First, the effect of the degree of polymerization (*X*_A_) on the parameters *a* and *ρ*_*0*_ was considered. To this end, the parameters are determined by applying the fitting procedure described in Part II‐3 to PDMS with different degrees of polymerization ($X_{A}=9,25,80,151,184$ and 230). For each polymer, Tait's equations used to reproduce the PVT experimental data over the intervals 25–70 °C and 0–900 bar are provided in Table S1, Supporting Information. It should be noted that the Tait's parameters are the same for $X_{A}=151,184$ and 230.^[^ ^29^ ^]^ In Figure S2, Supporting Information, experimental PVT data for PDMS at previous values of $X_{A}$ have been plotted and show that the density becomes chain length invariant for $X_{A}$ close to 100. For each polymer, the values of *a* and $\rho_{0}$ calculated over the interval 25–70 °C and for the two pressures 0 and 200 bar are given in **Table** **2**. In **Figure** **2**, the experimental density for $X_{A}=184$ (obtained through the Tait's parameters $V\left(0,T\right)=1.00576\;+\;0.993072\;\times \;10^{-3}t-0.078064\;\times \;10^{-6}t^{2}\;\text{and}\;B\left(T\right)=885\times \exp\left(-6.100\times 10^{-3}t\right)$, see Table S1, Supporting Information) and the theoretical density, obtained with $a=-1.557\times 10^{-20}J$ and $\rho_{0}=9.310\times 10^{27}{\text{ m}}^{-3}$ leading to the best fit, are plotted. The same study performed for all studied polymerization degrees shows that the parameters *a* and $\rho_{0}$ also converge to $a=-1.557\times 10^{-20}J$ and $\rho_{0}=9.310\times 10^{27}m^{-3}$ when $X_{A}$ increases. Over the total$X_{A}$ range studied, the variation is of the order of 3% for *a* and 6% for $\rho_{0}$, whereas the variation is less than 1‰ for *a* and 5‰ for $\rho_{0}$once the oligomer/polymer transition has been passed. These results suggest that *a* and ρ_0_ are independent of the degree of polymerization for $X_{A}$ above around 80 repeat units, however below this value, the polymer to oligomer transition affects both parameters.

**Table 2:** Values of *a* and $\rho_{0}$ determined by considering different degrees of polymerization for PDMS.

*X*_A_	*a* (10^−20^ J)	*ρ*_0_ (10^27^ m^−3^)	$\sqrt{\theta }\left(10^{-3}{\text{g cm}}^{-3}\right)$
9	−1.509	8.775	2.6
19	−1.536	8.913	2.5
25	−1.548	9.117	2.3
80	−1.555	9.262	2.3
151	−1.556	9.311	2.0
184	−1.557	9.310	2.0
230	−1.557	9.310	2.0

#### 2.2.2 Computing Surface Pressure—Mean Area Per Repeat Unit π=f(A) Isotherms

The variables involved in the thermodynamic model were then translated into the parameters involved in the film at the air–water interface. In bulk, the volume per repeat unit is given by $\frac{V}{N_{A}}=\frac{N}{\rho_{0}N_{A}}=\frac{1}{\rho_{0}\phi }$. For a given temperature *T*, it is possible to calculate for each pressure P the value of *ϕ* for which $\frac{\partial G}{\partial N}=0$ and deduce $\frac{1}{\rho_{0}\phi }$. Thus, by introducing the thickness *e* of the film then the mean area per repeat unit *A* can be written as $A=\frac{1}{e\rho_{0}\phi }$, while the surface pressure π can be expressed dimensionally as π=P×e. Based on the parameters of the thermodynamic model, the surface pressure–area isotherms of monolayers can be described by computing π=f(A).

**Figure 3:**
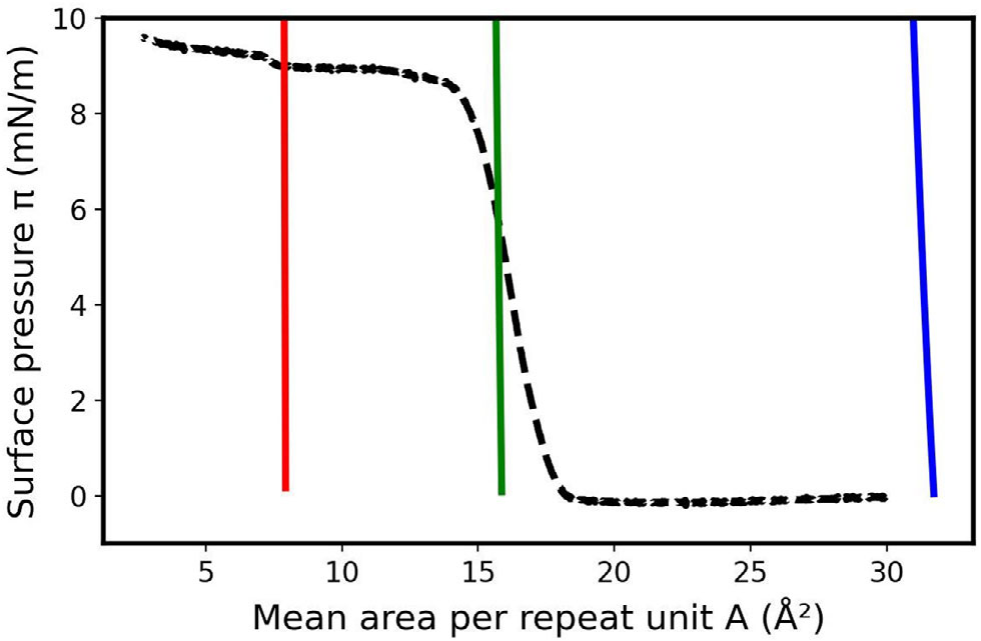
Comparison between the experimental isotherm at 20 °C of a PDMS (*X*_A_ = 184) monolayer (black line), and the theoretical ones obtained using the 3D thermodynamics parameters of the PDMS, that $a=-1.57\times 10^{-20}J$ and $\rho_{0}=9.29\times 10^{27}m^{-3}$, and different thicknesses equal to 4 Å (blue line), 8 Å (green line), and 16 Å (red line).

**Figure** **3** shows the π=f(A) isotherms computed for three constant thicknesses of the monolayer, *e* = 4, 8, and 16 Å, compared to the experimental curve for a monolayer of PDMS with *X*_A_ = 184. The aim is to describe the condensed phase experimentally observed from the lift‐off at around 18 Å^2^, where the surface pressure starts to increase, until the surface pressure plateau is evidenced at 9 mN m^−1^, as already reported in the literature.^[^ ^30^, ^31^, ^32^ ^]^

There is a good agreement between the mean surface densities of the experimental curve and the theoretical one obtained for e=8Å; however, the slope of the theoretical curve is incorrect. In the model proposed here, the slope of the theoretical isotherm is only controlled by *ϕ*. Indeed, $A=\frac{1}{e\rho_{0}\phi }$ and since *e* and $\rho_{0}$ are constant, *A* is proportional to 1/ϕ. However, as π varies from 1 to 7 mN m^−1^ in the condensed phase of this isotherm, the pressure *P* equal to π/e varies from 12.5 to 87.5 bar which corresponds to a variation in *ϕ* from ≈0.849  to ≈0.857, and therefore, the theoretical isotherm is almost vertical. Furthermore, during compression, the thickness of the monolayer logically varies from a value close to zero (not measurable in the gaseous state) to a measurable value of a condensed monolayer. This effect is well described by the model since the theoretical mean area per repeat unit decreases from more than 30 Å^2^ to less than 10 Å^2^ when the chosen thickness increases from 4 to 16 Å. The thickness of the monolayer depends, as expected and observed experimentally, on the surface pressure  π. To account for this phenomenon, the thickness *e* was assumed to increase linearly with the surface pressure π, as e=απ+β where *α* ($10^{-7}{\text{Pa}}^{-1}$) and *β* (Å) are adjustable parameters. Since the monolayer remains in a homogenous dense phase over the pressure range used for fitting, and the isotherm is approximately linear in this regime, we chose to model the thickness as a first‐order (linear) function of surface pressure. This is a phenomenological assumption, valid over a limited pressure range where no structural or phase transitions occur. The relation between the pressure *P* of the thermodynamic model and the surface pressure π is then given by

(2) π=P×(απ+β)

that is to say

(3) $$\pi =\frac{\beta P}{\left(1-\alpha P\right)}$$

**Figure 4:**
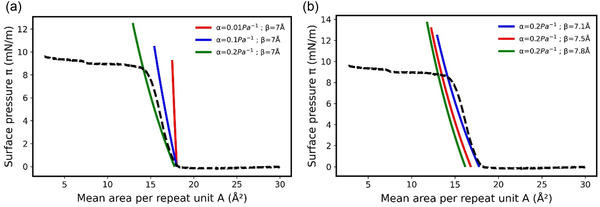
Comparison between the experimental isotherm of a PDMS monolayer (dashed line) at 20 °C for $X_{A}=184$, and the theoretical one (solid red line) obtained with $a=-1.57\times 10^{-20}J$ and $\rho_{0}=9.29\times 10^{27}m^{-3}$ and a monolayer thickness of e=απ+β with a) a constant value for béta = 7 angstrom and alpha varies and b) a constant value for alpha = 0.2 Pa^−1^ and béta varies.

To better understand the physical significance of *α* and *β*, these two parameters were independently varied to determine their effect on the resulting theoretical isotherm (**Figure** **4**).

**Figure 5:**
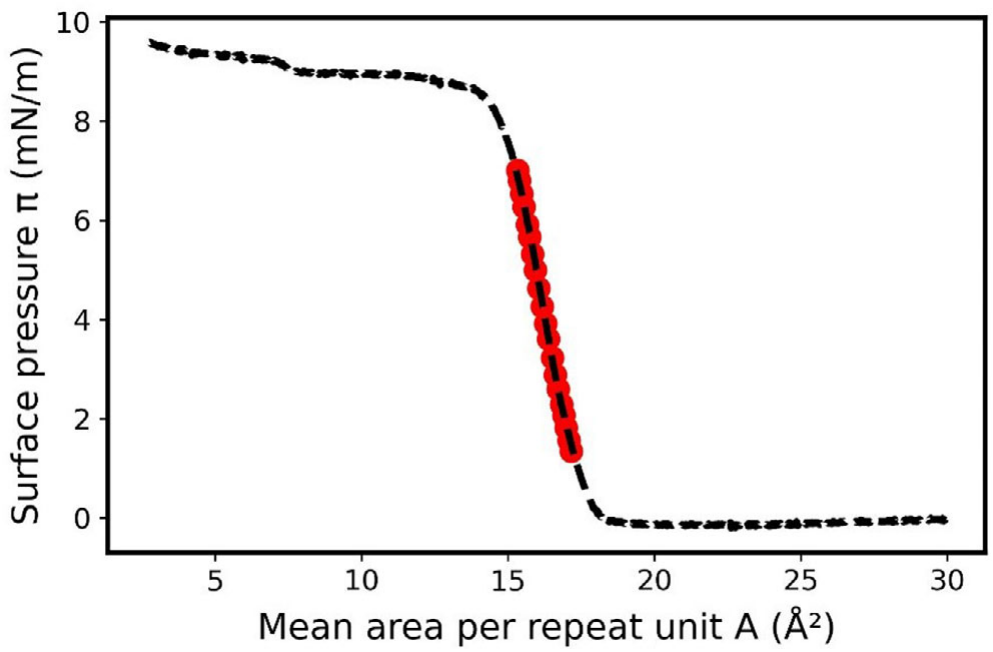
Comparison of experimental (dashed line) and theoretical (red circles) surface pressure–area isotherm of PDMS at 20 °C for $X_{A}=184$ between 1 and $7{\text{mN m}}^{-1}$. The theoretical curve was obtained with $a=-1.57\times 10^{-20}J$ and $\rho_{0}=9.29\times 10^{27}m^{-3}$, considering e=απ+β where $\alpha =0.15\times 10^{-7}{\text{Pa}}^{-1}$ and β=7.17Å.

*α* mainly affects the slope of the isotherm, while β is more representative of the mean area per repeat unit at zero surface pressure. These effects were expected since $A=\frac{1}{\rho_{0}\phi \left(\alpha \pi +\beta \right)}$. Then when π=0, the position of the isotherm is given by $A=\frac{1}{\rho_{0}\phi \beta }$, so the larger *β*, the more the isotherm translated toward smaller values of *A*. On the other hand, the closer *α* is to 0, the more the monolayer thickness is constant, i.e. the isotherm is vertical, as shown previously. The values of *α* and *β* can be assessed from the linear region (without phase transition) of the experimental compression isotherm. To do this, at a fixed temperature, the values ofα and *β* were varied, and for each pair, the corresponding values of *π* and *A* were calculated. Then, similarly to the determination of *a* and $\rho_{0}$, the pair that yields the smallest error between the theoretical and experimental compression curves was determined as follows

(4) $$\Gamma \left(\alpha ,\beta \right)=\frac{1}{N}\sum_{i=1}^{N}{\left(A_{\text{exp}}-A_{\text{th}}\right)}^{2}$$

where *N* is the number of pressures considered. This approach allows for a good fitting of the isotherm. For instance, the theoretical compression isotherm of PDMS was calculated between 1 and $7{\text{mN m}}^{-1}$ using the parameters $\alpha =0.15\times 10^{-7}{\text{Pa}}^{-1}$ and β=7.17Å, providing the best fit, and compared to the experimental isotherm (**Figure** **5**).

From the values of *α* and *β*, it is possible to determine the thickness of the monolayer for any surface pressure value since e=απ+β. In this case, the theoretical thickness of 7.9Å was found at $5{\text{mN m}}^{-1}$, which is very close to the experimental thickness determined from neutron reflectivity measurements at e=8Å (measured at $5{\text{mN m}}^{-1}$), confirming the validity of the model proposed.^[^ ^24^ ^]^ Finally, the predictions concerning both the surface pressure–area isotherm and the thickness of the PDMS monolayer are correct.

### 2.3 Application to Other Polymer Monolayers

The monolayers of PMMA, PTHF, and 1,2‐PB at the air–water interface were then modeled.

#### 2.3.1 PMMA Monolayer

**Figure 6:**
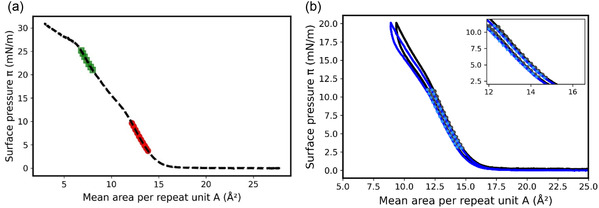
a) Comparison of experimental (dashed line) and theoretical (green squares and red circles) surface pressure–area isotherms of PMMA at 20 °C. The fit is obtained with $\alpha =0.23\times 10^{-7}{\text{Pa}}^{-1}$ and β=9.20 Å on the range 3–$10{\text{mN m}}^{-1}$and with $\alpha =0.73\times 10^{-7}{\text{Pa}}^{-1}$and $\beta =1.97\;Å\;\text{on the range}\;21\;\text{to}\;25\;{\text{mN m}}^{-1}$. b) Two successive compression–expansion hysteresis cycles at 20 mN m^−1^ of the PMMA monolayer, along with the fits of the compression and expansion curves on the range 3–$10\;{\text{mN m}}^{-1}$.

Some polymers undergo phase transitions during compression. Therefore, it is important to verify that this model is still applicable in different regions. To do this, we applied the same methodology to PMMA, which exhibits a phase transition at around 13 mN m^−1^ (**Figure** **6**). As before, the *a* and *ρ*_*0*_ parameters were evaluated from the Tait's equation of PMMA (where $V\left(0,T\right)=0.8254+2.838310^{-4}t+7.79210^{-7}t^{2}$ and $B\left(T\right)=2875\;\;\text{exp}\left(-4.146\times 10^{-3}t\right)$) to be equal to *a* = −2.49.10^−20^ J and *ρ*_0_ = 7.87.10^27^ m^−3^ while *m*_Φ_ = 100.1 g mol^−1^ (the comparison between experimental and theoretical densities are reported in Supporting Information (Figure S3, Supporting Information)). The theoretical compression isotherm was determined, on one hand, between 3 and 10 mN m^−1^ and, on another hand, between 21 and 25 mN m^−1^ (Figure 6). The *α* and *β* parameters used to calculate the monolayer thicknesses in these two phases are reported in **Table** **3**.

**Table 3:** Values of *α* and *β* determined for the different surface pressure domains and the corresponding thicknesses *e* of the PMMA monolayer.

Pressure domain	$\alpha \left[10^{-7}{\text{Pa}}^{-1}\right]$.	β [Å]	*e* [Å]
3–10 [mN m^−1^]	0.23	9.20	10.4 at 5 [mN m^−1^]
21–25 [mN m^−1^]	0.73	1.97	18.0 at 22 [mN m^−1^]

The theoretical compression isotherm is in very good agreement with the experimental one in both surface pressure ranges considered, each corresponding to a distinct dense phase. This demonstrates that the developed model can be applied regardless of the dense phase in which the monolayer is found. In addition, the theoretical thickness of 18Å at 22 mN m^−1^ is in perfect agreement with the value measured by X‐ray reflectivity for a PMMA monolayer transferred onto a solid substrate, further confirming the relevance of the proposed model.^[^ ^33^ ^]^ These findings indicate that, although the model does not explicitly account for phase transitions, it can nevertheless accurately describe both low‐ and high‐pressure dense phases, each characterized by distinct surface densities and compressibilities, yielding significantly different layer thicknesses that are consistent with experimental observations.

We further investigated the PMMA monolayer by studying both experimentally and theoretically possible hysteresis phenomena during compression–expansion cycles. Three successive compression (up to 20 mN m^−1^) and expansion (down to 0 mN m^−1^, 25 Å^2^ per repeat unit) cycles were monitored (Figure S4, Supporting Information and Figure 6b for the first two cycles). First, all expansion curves are slightly shifted to smaller areas compared with the compression ones; however, the maximum shift reaches only 0.7 Å^2^ in the high‐pressure region. A slight hysteresis is visible between the first and second compression curves, with a maximum difference of less than 0.5 Å^2^. In contrast, the second and third cycles are superimposed, indicating the stability of the polymer layer. These observations suggest minor polymer rearrangement during the first cycle and weak relaxation phenomena. The isotherms for both compression and expansion were fitted for all three cycles, and the evolution of the monolayer thickness at 5 mN m^−1^ throughout the cycling process was evaluated. The resulting values of *e* = 9.9 Å during the first compression and 10.2 Å during the first expansion are consistent with the slight hysteresis observed during the first compression–expansion cycle. The values obtained during the second (9.9/10.2 Å) and third (10.0/10.2 Å) cycles also remain consistently very close to those obtained during the first cycle.

This study demonstrates that the model effectively characterizes thickness variations during compression–expansion cycles, which may reflect different phenomena depending on the polymer, such as structural rearrangements, kinetic effects, or partial dissolution of the polymer into the water subphase. However, in the present case, the thickness variation is negligible. Overall, these results support the robustness of the fitting procedure, which enables the study of hysteresis phenomena.

#### 2.3.2 PTHF Monolayer

**Figure 7:**
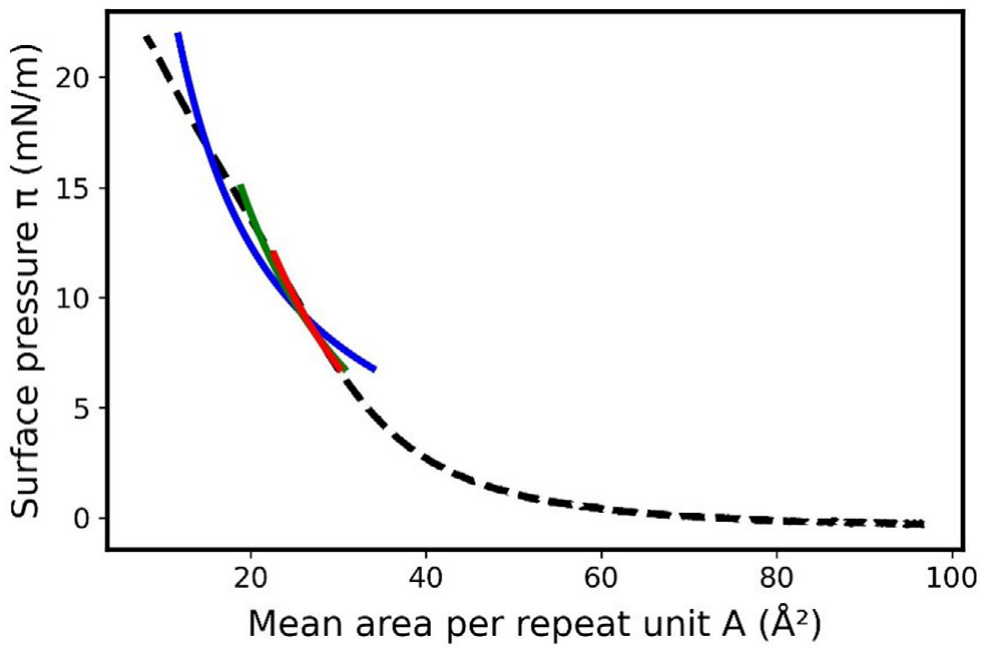
Comparison of experimental (dashed line) and different theoretical surface pressure–area isotherms at 20 °C for a PTHF monolayer, obtained using several fitting ranges: $7\;\text{to}\;22\;{\text{mN m}}^{-1}$ in blue, $7\;\text{to}\;15\;{\text{mN m}}^{-1}$ in green, and $7\;\text{to}\;12\;{\text{mN m}}^{-1}$ in red. All the theoretical curves were obtained with $a=-1.97\times 10^{-20}J$, $\rho_{0}=9.18\times 10^{27}m^{-3}$, and $X_{A}=1000$.

The same methodology was applied to a PTHF monolayer, which does not exhibit any phase transition over a wide range of surface pressure (**Figure** **7**). The *a* and ρ_0_ parameters were evaluated from the Tait's equation of PTHF (where $V\left(0,T\right)=1.0043\times \text{exp}\left(6.691\times 10^{-4}t\right)$ and $B\left(T\right)=1786\times \text{exp}\left(-4.223\times 10^{-3}t\right)$) to be equal to *a* = −1.97.10^−20^ J and ρ_0_ = 9.18.10^27^ m^−3^ while *m*_Φ_ = 72.1 g mol^−1^ (the experimental and theoretical densities are shown in Supporting Information (Figure S3, Supporting Information)). The *α* and *β* parameters used to calculate the monolayer thicknesses in these different surface pressure ranges are reported in **Table** **4**.

**Table 4:** Values of *α* and *β* determined for the different surface pressure ranges and corresponding thicknesses *e* of the PTHF monolayer.

Pressure domain	α [10^−7^ Pa^−1^]	β [Å]	*e* [Å] at 7 [mN m^−1^]	*e* [Å] at 10 [mN m^−1^]	*e* [Å] at 12 [mN m^−1^]
7–22 [mN m^−1^]	0.79	0.43	2.9	5.1	5.9
7–15 [mN m^−1^]	0.30	1.96	3.5	5.0	5.6
7–12 [mN m^−1^]	0.26	2.32	3.6	4.9	5.4

Depending on the chosen pressure range, the *α* and *β* values vary by approximately 70–75%. However, the thickness values only vary by less than 1 Å. Thus, even though the fits are not very good, the variation in thickness depending on the fitting range is close to the experimental uncertainty.

#### 2.3.3 1,2‐polybutadiene Monolayer

Finally, the same methodology was applied to a 1,2‐polybutadiene (1,2‐PB). When spread at the air‐water interface, this polymer progressively oxidizes until a stationary state is reached and a compression isotherm is measured.^[^ ^34^ ^]^ Indeed, the unoxidized polymer does not form any monolayer at the air–water interface, probably due to its hydrophobic character. Thus, due to the modified molecular structure of the 1,2‐PB at the air–water interface, the interactions between the repeat units characterized by *a* parameter and the maximum density characterized by *ρ*_*0*_ should therefore be affected. Thus, the proposed model should no longer apply correctly.

**Figure 8:**
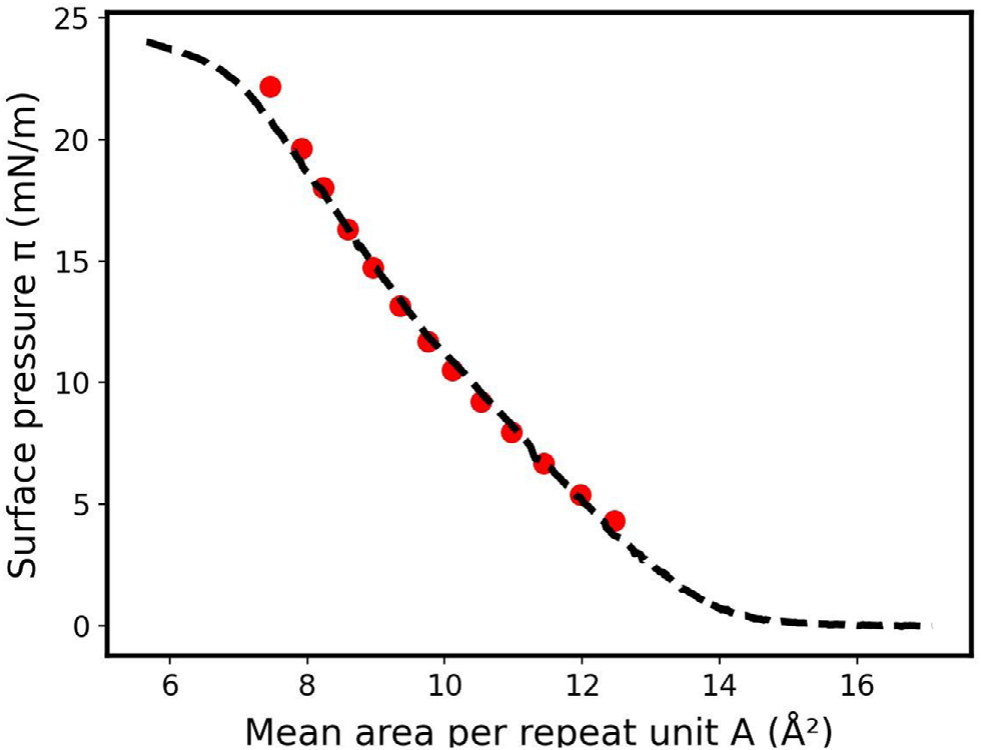
Comparison of experimental (dashed line) and theoretical (red circles) surface pressure–area isotherm of 1,2‐PB at 20 °C between 4 and 22 mN m^−1^ . The theoretical curve was obtained with $a=-1.81\times 10^{-20}J$ and $\rho_{0}=11.36\times 10^{27}m^{-3}$, considering e=απ+β where $\alpha =0.30\times 10^{-7}{\text{Pa}}^{-1}$ and β=6.78Å.

The *a* and ρ_0_ parameters were evaluated to be equal to *a* = −1.81.10^−20^ J and *ρ*_0_ = 11.36.10^27^ m^−3^ while m_Φ_ = 54.6 g mol^−1^ (the experimental and theoretical densities are shown in Supporting Information (Figure S3, Supporting Information)). The theoretical compression isotherm was then determined between 4 and 22 mN m^−1^ and compared to the experimental one (**Figure** **8**). Despite the large range of surface pressure, a good agreement is obtained.

The monolayer thicknesses were calculated at different surface pressures and compared to the experimental ones determined from neutron reflectivity measurements (**Table** **5**).^[^ ^35^ ^]^

**Table 5:** Values of calculated and experimental thicknesses for a 1,2‐PB monolayer at different surface pressures.

Surface pressure	Calculated thickness [Å]	Experimental thickness [Å]
5 [mN m^−1^]	8.2	9
10 [mN m^−1^]	9.8	13 (dense upper layer)‐ + 17 (hydrated bottom layer)
15 [mN m^−1^]	11.3	14 (dense upper layer)‐ + 28 (hydrated bottom layer)

Experimentally, it was observed that at 5 mN m^−1^, the 1,2‐PB forms a thin dense layer while beyond 10 mN m^−1^, a highly hydrated layer grows underneath this dense layer. It was attributed to the increased hydrophilic character of the polymer because of oxidation, leading to some PB chains extending towards the water upon monolayer compression.

It appears that the thickness calculated at 5 mN m^−1^ is online with the experimental one. However, the thicknesses determined at 10 and 15 mN m^−1^ significantly move away from the experimental values. Even the thickness of the upper dense layer cannot be reproduced, with values 25 and 19% higher than the theoretical ones at 10 and 15 mN m^−1^, respectively. These significant differences are likely to be due to the modification of the chemical structure of the 1.2‐PB, which oxidizes and therefore becomes more hydrophilic. The *a* and *ρ*_*0*_ parameters are no longer correct. This clearly shows the importance of these parameters to assess reliable thicknesses of the monolayer through the model.

## 3 Discussion

**Figure 9:**
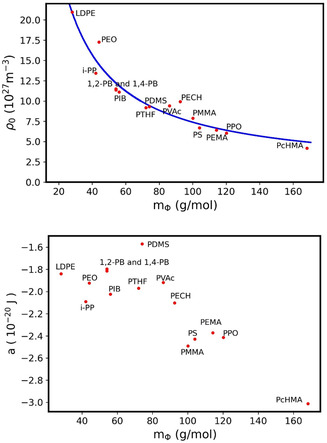
Evolution of $\rho_{0}$ (top) and *a* (bottom) as a function of molecular mass of a repeat unit $m_{\phi }$ for the various polymers considered: LDPE (low density – polyethylene), i‐PP (isopolypropylene), PEO (polyethyleneoxide), 1,2‐PB (1,2‐polybutadiene), 1,4‐PB (1,4‐polybutadiene), PIB (polyisobutylene), PTHF (polytetrahydrofuran), PDMS (polydimethylsiloxane), PVAc (polyvinyl acetate), PECH (polyepichlorhydrine), PMMA (polymethylmethacrylate), PS (polystyrene), PEMA (polyethylmethacrylate), PPO (polyphenylene oxide), and PcHMA (polycyclohexylmethacrylte). $\rho_{0}$ was fit as a function of $m_{\phi }$, as $\rho_{0}={1.215\times 10}^{+27}+\frac{6.42510^{+29}}{m_{\phi }}-\frac{2.57410^{+30}}{m_{\phi }^{2}}$.

Finally, the first results on the effect of *X*_A_ on the values of *a* and *ρ*_*0*_ have suggested that *a* is significantly dependent on chain length, whereas *ρ*_*0*_ is a more characteristic parameter of the repeat unit under consideration. To verify this assertion, the *a* and ρ_0_ parameters were determined for various polymers using the PVT data computed through Tait's equations (Table S1, Supporting information). The polymers which were investigated and the corresponding *a* and ρ_0_ parameters are also given in Supporting Information (Table S2, Supporting information). The variation of $\rho_{0}$ as a function of the molar mass of the polymer repeat unit under consideration $m_{\phi }$ is shown in **Figure** **9**.

As the molar mass of the repeat unit decreases, $\rho_{0}$ increases, which is consistent with the fact that $\rho_{0}$ represents the maximum repeat unit compaction that can be put into $1m^{3}$. It should be noted that 1,2‐PB and 1,4‐PB have very similar $\rho_{0}$ values, in good agreement with the assumption that it is a characteristic parameter of the repeat unit. Figure 9 also shows a fit of $\rho_{0}$versus $m_{\phi }$ according to a law of the form $a+\frac{b}{m_{\phi }}+\frac{c}{m_{\phi }^{2}}$. The best fit is obtained for $a=1.215\times 10^{27}m^{-3}$, $b=6.435\times 10^{29}g/\left(\text{mol}\cdot m^{3}\right)$ and $c=-2.575\times 10^{30}g^{2}/\left({\text{mol}}^{2}\cdot m^{3}\right)$. The choice of this fit was motivated solely by the need to minimize the squared deviation and not by any physical arguments. However, the polymers with a $m_{\phi }$ value greater than 100 g mol^−1^ considered in this study (i.e., PEMA, PS, PPO, PMMA, and PcHMA) bear a side group. In particular, the difference between PEMA, PMMA, and PcHMA is the side group connected to the polymethacrylate main chain, which is much larger in the case of PcHMA and could lead to stronger interactions or steric hindrance. In parallel, the effect of $m_{\phi }$ on the interaction parameter *a* was also investigated (Figure 9). As expected, *a* is independent of $m_{\phi }$ as long as $m_{\phi }$ is less than 100 g mol^−1^. For example, PEO and PTHF have very close *a* values (−1.923.10^−20^ and −1.970.10^−20^ J, respectively). Thus, increasing the length of the main chain repeat unit has no effect on the *a* parameter.

Regarding the determination of thickness, the various examples presented show that the evaluation made from the model agrees with experimental measurements. However, it is possible to obtain a very simple approximation of the thickness *e* without following the entire previous procedure. Indeed, by inverting the expression of *A* as a function of *e* ($e=1/\left(A\rho_{0}\phi \right)$), as *ϕ* is typically of the order of 0.85−0.90 for all the polymers studied over the pressure range of interest in monolayers. This expression is particularly interesting as $\rho_{0}$ can be obtained using the fit curve given in Figure 9. For instance, for PDMS monolayer, the PDMS close‐packing parameter would be approximatively $\rho_{0}\approx 9.43\times 10^{27}m^{-3}$. Thus, at $\pi =5{\text{mN m}}^{-1},A\approx 16{Å}^{2}$ and ϕ=0.85 lead finally to a thickness of e=7.8Å which is close to the experimental value (e=8Å).

## 4 Conclusion

It has been demonstrated that utilizing a thermodynamic model with no adjustable parameters enables the implementation of the polymer monolayer thickness as a function of surface pressure, e(π), to be easily achieved. To accomplish this, the interaction parameter *a* between repeat units of any polymer and the number of repeat units per unit volume at close packing (infinite pressure) ρ_0_ were determined using the thermodynamic model based on Tait's equation for the considered polymer. Subsequently, a transition from 3D to 2D (isothermal) was made by converting pressure to surface pressure and volume density to surface area. Finally, the experimental compression isotherm could be fitted by considering that the monolayer thickness linearly increases with surface pressure.

Finally, this approach was applied to several monolayers of different polymers, four of which were presented in this work. For three of them, we found the thickness of the monolayer that had been experimentally measured beforehand. The disagreement for the fourth one (1,2‐PB) because of its chemical modification upon spreading at the air–water interface reinforces the relevance of the model parameters to describe monolayers at the air–water interface. Although the physics of such monolayers is highly complex, the model provides very good results when the chemical structure is stable. This work will be further extended to amphiphilic block copolymer Langmuir films that undergo a mushroom‐to‐brush phase transition, in which the two states exhibit markedly different surface densities and compressibilities. Extending the model to describe monolayers across various first‐order phase transitions, such as the gas‐to‐liquid transition, would also represent a valuable development. These results will ultimately be applied to predict phase separation in polymer blends formed at the air–water interface.

## 5 Experimental Section

5.1

5.1.1

##### Polymers

PTHF (*M*_W_ ≈  2900 g mol^−1^), atactic PMMA (*M*_w_ ≈ 120 000 g mol^−1^, viscosity 250–560 cps), and 1,2‐poly(butadiene) (PB) (containing 90% of 1,2‐PB and 10% of 1,4‐PB, *M*_w_ = 5 500 g mol^−1^ and a polydispersity index of 1.5 determined by size exclusion chromatography) were bought from Sigma‐Aldrich. PDMS (*M*_W_ = 13 650 g mol^−1^) was purchased from ABCR.

##### Preparation of the Monolayer at the Air–Water Interface

Monolayers were prepared in a medium‐sized Langmuir**–**Blodgett trough from KSV Nima company equipped with a Wilhelmy balance for surface pressure measurement. The trough was filled with an ultra‐pure water subphase (resistivity of 18.2 MΩ cm, Millipore Simplicity) at a temperature set to 20 °C using a water circulator connected to a thermal bath. The polymers were dissolved in dichloromethane (CH_2_Cl_2_, ≥99%, GPR Rectapur, VWR chemicals) at a concentration of 0.4 mg mL^−1^. An appropriate volume of the polymer solution was spread on the water subphase using a microsyringe. The initial mean area per repeat unit *A* was set to 100 Å^2^ for PTHF and 30 Å^2^ for the other polymers. After allowing 10 minutes for solvent evaporation, the movable barrier was used to reduce the available area *A* at a compression speed of 10 mm^2^ min^−1^. Simultaneously, the surface pressure π was recorded, leading to π=f(A).

It should be emphasized that to ensure the reproducibility of the π=f(A) isotherms and stability over time of the different polymer monolayer states, the compression isotherms were recorded at a low compression speed (10 mm^2^ min^−1^) and using various experimental conditions including different quantities of polymer deposited on the water surface and spreading solutions at different concentrations.

##### Thermodynamic Model

The used model, which is extended from the model published in 2001,^[^ ^36^ ^]^ was first developed to describe the thermodynamics of compressible polymer blends under controlled pressure *P* and temperature *T* conditions.^[^ ^23^ ^]^ This model, which does not explicitly account for the coordination number (because we do not consider a “network” as in Flory's model), gives for a pure polymer the Gibbs free energy *G* as follows

(5) $$G\left(N,N_{A},P,T\right)=a\frac{N_{A}^{3}}{N^{2}}+k_{B}T\frac{N_{A}}{X_{A}}\ln\left(\frac{N_{A}}{N}\right)-k_{B}TN_{A}\ln\left(1-\frac{N_{A}}{N}\right)+\frac{PN}{\rho_{0}}$$

where $N_{A}$ is the number of repeat units of polymer, and $X_{A}$ its degree of polymerization. Moreover, *N* is the dimensionless volume of the system. This parameter was introduced thanks to the close‐packing parameter $\rho_{0}$, which gives the density of repeat units at zero free volume in the system. Thus, the total volume of the system *V* was equal to $N/\rho_{0}$. The *a* parameter describes the interactions between repeat units. Thus, the first term of Equation (4) corresponds to internal energy, the second one to the translational entropy, which is the same as the one proposed in the classical Flory–Huggins model, due to the permutations at fixed configuration. An additional contribution to the entropy (the third term) was introduced to consider the free volume and then the mobility of each repeat unit in its surrounding volume. The last term corresponds to the term PVwritten as a function of the dimensionless volume *N*.

The equation of state of the pure polymer was obtained by computing the equation of state ∂G/∂N=0, which leads to

(6) $$-2a\phi^{3}+\frac{P}{\rho_{0}}-k_{B}T\left(\frac{\phi }{X_{A}}+\frac{\phi^{2}}{1-\phi }\right)=0$$

where the volume fraction of the polymer, $\phi =N_{A}/N$ depends on the pressure *P*, the temperature *T* but also the close packing parameter $\rho_{0}$ and the interaction energy *a*. Solving Equation (5), the volume fraction *ϕ* can be determined as a function of *a* and $\rho_{0}$ for a given $X_{A}$, pressure *P* and temperature *T*. It should be noted that for a dense phase, the parameter *ϕ* was of the order of 0.9. Equation (5) is very close to the equations of state obtained in the cell model developed by Prigogine^[^ ^37^, ^38^ ^]^ or the one derived in the Flory–Orwoll–Vrij model.^[^ ^29^, ^39^ ^]^ In addition, *a* and $\rho_{0}$ can be determined by fitting the experimental PVT data of the considered polymer since the theoretical density *ρ* can be deduced from the volume fraction *ϕ* through the expression

(7) $$\rho \left(P,T,a,\rho_{0}\right)=\frac{m_{\phi }\rho_{0}\phi \left(P,T,a,\rho_{0}\right)}{N_{A}}$$

where $N_{A}$ is the Avogadro number and $m_{\phi }$ is the molar mass of the repeat unit of polymer (g mol^−1^). To do so, for convenience, the Tait's equation was chosen to reproduce the experimental PVT data.^[^ ^30^, ^31^, ^40^, ^41^ ^]^ This equation is the empirical expression of the volume occupied by a given polymer as a function of pressure and temperature

(8) $$V\left(P,T\right)=V\left(0,T\right)\left(1-C.\text{ln}\left(1+\frac{P}{B\left(T\right)}\right)\right)$$

where the coefficient *C* is usually taken to be a constant equal to 0.0894, while V(0,T) and B(T) are empirical functions with nontrivial temperature‐dependent terms whose expressions depend on the polymer and enable the best fits to be obtained (Table S1, Supporting Information).^[^ ^32^ ^]^ While for most of the polymers, exponential expressions of V(0,T) and B(T) give the best fit of the experimental PVT data. For some other polymers, these expressions are polynomial. As far as we know, the difference does not relate to physical properties but only to the quality of the PVT data fit. The average deviation of Tait's equation compared to the experimental results typically remains close to the experimental uncertainty, which is smaller than 0.001 cm^3^ g^−1^. Thus, the experimental uncertainty and the uncertainty due to the Tait equation can be considered negligible compared with the error in fitting the PVT data with a thermodynamics model.

Thus, experimental density was determined from Tait's equation as

(9) $$\rho^{\exp}\left(P,T\right)=\frac{1}{V\left(P,T\right)}$$

Achieving accurate fits requires a sufficient amount of experimental data. To address this, we chose to use Tait's equation to generate additional data, even when the temperature at which the experimental π=f(A) isotherm was measured falls outside the range for which Tait's equation was originally developed. To evaluate the potential error introduced by this approach, we compared in Figure S1, Supporting Information, experimental densities of two PDMS samples calculated using Tait's equations. For the first one, *X*_A_ = 80 and the Tait's equation is valid in the 25–70 °C range, while for the second one, *X*_A_ = 68, and the Tait's equation is valid over the 30–323 °C range. The densities calculated using Tait's equation, valid over the 25–70 °C range, remain in good agreement with those measured over the 25–130 °C range. Similarly, the densities obtained from Tait's equation, valid over the 30–323 °C range, can reliably extend down to 25 °C. This comparison demonstrates that the extrapolation of Tait's equation beyond its original range introduces minimal error, supporting its use in this context.

Finally, for a given polymer, a temperature range {$T_{i}$} and a pressure one {$P_{j}$}, the difference between the experimental and the theoretical densities was estimated from the following quantity, which needs to be minimized

(10) $$\theta \left(a,\rho_{0}\right)=\frac{1}{N_{T}N_{P}}\sum_{i=1}^{N_{T}}\sum_{j=1}^{N_{P}}{\left(\rho \left(P_{j},T_{i},a,\rho_{0}\right)-\rho^{\text{exp}}\left(P_{j},T_{i}\right)\right)}^{2}$$

where $N_{T}$ and $N_{p}$ are the number of temperatures and pressures considered in the temperature {$T_{i}$} and pressure {$P_{j}$} ranges, respectively. The square root of the previous quantity can be interpreted as the average error of the model when fitting. It is then sufficient to find the values of *a* and *ρ*_0_ which minimize this quantity.

## Conflict of Interest

The authors declare no conflicts of interest.

## Supporting information

Supplementary Material

## Data Availability

The data that support the findings of this study are available from the corresponding author upon reasonable request.
