# Supplementary material for: Modeling Thermodynamic Behavior of Ultrathin Films: Comparison with Experiments
Source: Chemphyschem. 2025 Jul 16;26(17):e202500199. doi: 10.1002/cphc.202500199 (PMC12447126; doi:10.1002/cphc.202500199)
Supplement: Supplementary file 1 — Supplementary Material [file CPHC-26-e202500199-s001.pdf]

Modelling thermodynamic behavior of ultrathin films: comparison with experiments

Modibo Camara, Elian Masnada, Sophie Cantin, Odile Fichet*

CY Cergy Paris Université, LPPI, F95000 Cergy, France

Table SI-1: List of range of temperature and Tait's parameters for different polymers. In the expressions of $B(T)$ and $V(0,T)$, the temperatures noted t are expressed in degree Celsius, and the temperatures noted T are expressed in Kelvin. For all polymers, Tait's equations were used over a pressure range from 1 bar to 200 bar, which is within the validity range of the coefficients presented. Unless specified, data are from the reference Rodgers, P.A. (1993), Pressure–volume–temperature relationships for polymeric liquids: A review of equations of state and their characteristic parameters for 56 polymers. J. Appl. Polym. Sci., 48: 1061-1080. . Polymers for which surface pressure - area isotherms are studied in this work are shown in bold.

Polymer	molecular mass of a repeat unit (g/mol)	Temperature Range t (in °C))	X_A	$V(0,T)$ (cm^3/g)<br>$B(T)$ (bar)
LDPE<br>*low density* - Polyethylene	28.05	121-175		$V(0,T) = 1.1595 + 2.841 \times 10^{-4}t$<br>$B(T) = 2022 \times \exp(-5.243 \times 10^{-3}t)$
i-PP<br>*iso*-Polypropylene	42.08	170-297		$V(0,T) = 1.1606 \times \exp(6.7 \times 10^{-4}t)$<br>$B(T) = 1491 \times \exp(-4.177 \times 10^{-3}t)$
PEO<br>Polyethyleneoxide	44.05	88-224		$V(0,T) = 0.8766 \times \exp(7.087 \times 10^{-4}t)$<br>$B(T) = 2077 \times \exp(-3.947 \times 10^{-3}t)$
**PB (87% 1-2 and 13% 1,4)[1]**<br>**1,2-Polybutadiene**	**54.09**	$\mathbf{25 - 200}$	2400	$\boldsymbol{V(0,T) = 1.1094 + 0.6729 \times 10^{-3}t + 0.4470 \times 10^{-6}t^2}$<br>$\boldsymbol{B(T) = 1750 \times exp(-4.538 \times 10^{-3}t)}$
1,4-PB<br>1,4-Polybutadiene	54.09	$4 - 55$		$V(0,T) = 1.0970 \times \exp(6.600 \times 10^{-4}t)$<br>$B(T) = 1777 \times \exp(-3.593 \times 10^{-3}t)$
PIB	56.11	53-110		$V(0,T) = 1.075 \times \exp(5.651 \times 10^{-4}t)$<br>$B(T) = 2003 \times \exp(-4.329 \times 10^{-3}t)$

[1] Yi, Y. X., & Zoller, P., An experimental and theoretical study of the PVT equation of state of butadiene and isoprene elastomers to 200 C and 200 MPa. *Journal of Polymer Science Part B: Polymer Physics*, **1993**, *31*(7), 779-788

Polyisobutylene				
**PTHF**<br>**Polytetrahydrofuran**	**72.11**	**62-166**		$V(0,T) = 1.0043 \times \exp(6.691 \times 10^{-4} t)$<br>$B(T) = 1786 \times \exp(-4.223 \times 10^{-3} t)$
	**74.11**	**25-70**	9	$V(0,T) = 1.07411 + 1.23519 \times 10^{-3} t + 0.452937 \times 10^{-6} t^2$<br>$B(T) = 728 \times exp(-7.444 \times 10^{-3} t)$
	**74.11**	**25-70**	19	$V(0,T) = 1.05319 + 1.10547 \times 10^{-3} t + 0.362133 \times 10^{-6} t^2$<br>$B(T) = 812 \times \exp(-6.865 \times 10^{-3} t)$
**PDMS** [2]<br>**Polydimethylsiloxane**	**74.11**	**25-70**	25	$V(0,T) = 1.02783 + 1.07383 \times 10^{-3} t - 0.060039 \times 10^{-6} t^2$<br>$B(T) = 847 \times exp(-6.487 \times 10^{-3} t)$
	**74.11**	**25-70**	80	$V(0,T) = 1.01000 + 1.04600 \times 10^{-3} t - 0.307921 \times 10^{-6} t^2$<br>$B(T) = 874 \times exp(-5.985 \times 10^{-3} t)$
	**74.11**	**25-70**	151<br>184<br>230	$V(0,T) = 1.00576 + 0.993072 \times 10^{-3} t - 0.078064 \times 10^{-6} t^2$<br>$B(T) = 885 \times exp(-6.100 \times 10^{-3} t)$
**PDMS**[2]<br>**Polydimethylsiloxane**	**74.11**	$30 - 323$	68	$V(0,T) = 0.8146 + 0.5578 \times 10^{-3} T + 0.5574 \times 10^{-6} T^2$<br>$B(T) = 4827.3 \times exp(-6.09 \times 10^{-3} T)$

[2] Fakhreddine, Y. A., & Zoller, P., The equation of state of a polydimethylsiloxane fluid. *Journal of Applied Polymer Science*, **1990**, *41*(5-6), 1087-1093

PVAc<br>Polyvinylacetate	86.09	35-100		$V(0,T) = 0.82496 + 5.82 \times 10^{-4} t + 2.94 \times 10^{-7}\ t^2$<br>$B(T) = 2049 \times \exp(-4.346 \times 10^{-3} t)$
PECH<br>Polyepichlorhydrin	92.52	60-140		$V(0,T) = 0.7216 \times \exp(5.825 \times 10^{-4} t)$<br>$B(T) = 2383 \times \exp(-4.171 \times 10^{-3} t)$
**PMMA**<br>**Polymethylmethacrylate**	**100.12**	**114-159**		$\boldsymbol{V(0,T) = 0.8254 + 2.8383 \times 10^{-4} t + 7.792 \times 10^{-7}\ t^2}$<br>$\boldsymbol{B(T) = 2875 \times \exp(-4.146 \times 10^{-3} t)}$
PS<br>Polystyren	104.1	115-196		$V(0,T) = 0.9287 \times \exp(5.131 \times 10^{-4} t)$<br>$B(T) = 2169 \times \exp(-3.319 \times 10^{-3} t)$
PEMA<br>Polyethylmethacrylate	114.14	113-161		$V(0,T) = 0.8614 \times \exp(7.468 \times 10^{-4} t)$<br>$B(T) = 2609 \times \exp(-5.356 \times 10^{-3} t)$
PPO<br>Poly(phenylene oxide)	120.16	203-320		$V(0,T) = 0.78075 \times \exp(2.151 \times 10^{-5} (t + 273.15)^{3/2})$<br>$B(T) = 2609 \times \exp(-5.356 \times 10^{-3} t)$
PcHMA<br>Polycyclohexylmethacrylate	168.23	123-198		$V(0,T) = 0.8793 + 4.0504 \times 10^{-4} t + 7.774 \times 10^{-7}\ t^2$<br>$B(T) = 2952 \times \exp(-5.22 \times 10^{-3} t)$

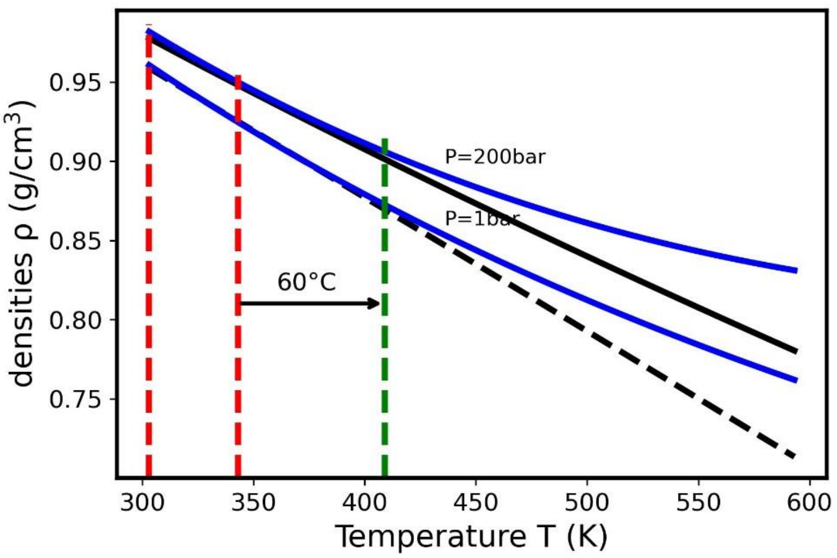

Figure SI-1 : Comparison of 3D PVT data obtained for two PDMS: the first one (in blue) corresponds to X_A=80 and Tait's parameters are valid over the 25-70 °C interval; the second one (in black) corresponds to X_A=68 and Tait's parameters are valid over the 30-323 °C interval. This figure shows that the validity intervals of the Tait's parameters can be widely extended.

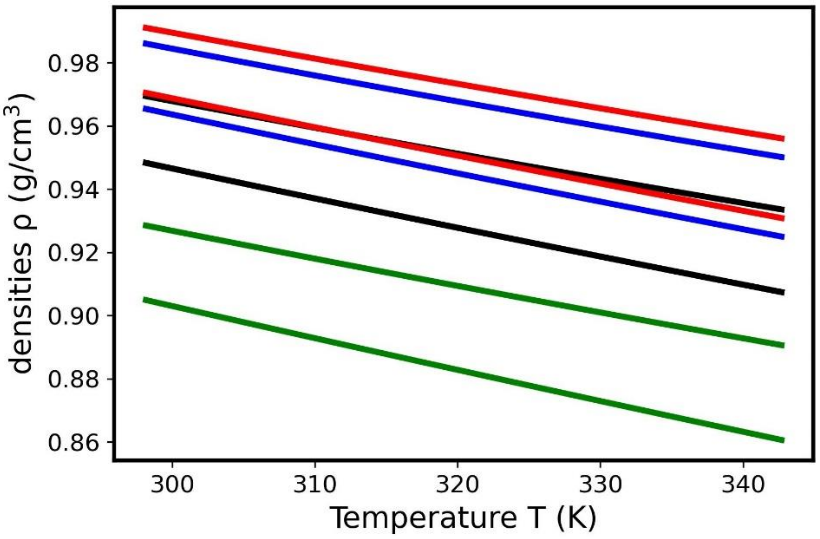

Figure SI-2 : Experimental PDMS density for different degrees of polymerization: $X_A = 9$ *(green),* 25 *(black),* 80 *(blue),* 151 *and* 230 *(red). For each degree of polymerization, the low density curve corresponds to P=1 bar, while the highest density curve corresponds to P=200 bar. These experimental data are obtained from Tait's equations (see Table SI-1). For* $X_A = 151$ *and* 230 *the Tait's parameters are identical, showing that the PVT data for these two degrees of polymerization are equal. This figure shows that PVT data become independent of chain length for* X_A *close to* 100.

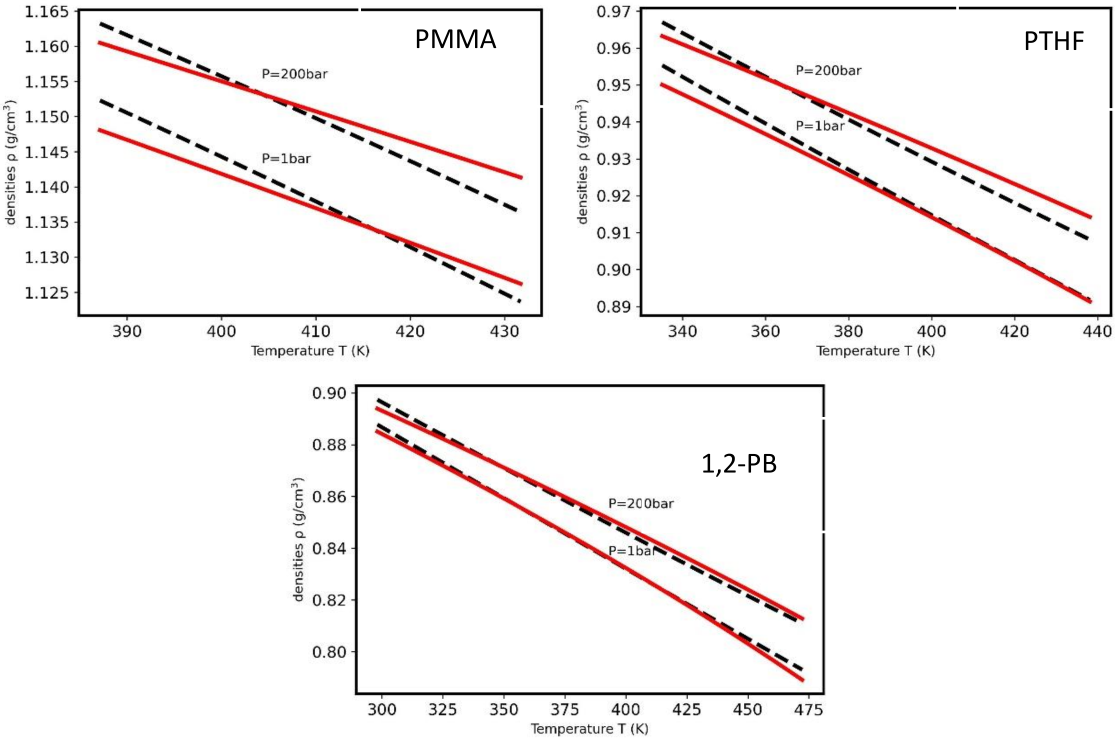

Figure SI-3: Comparison between the experimental density (dashed black curves) and the best fit (solid red curves) at 1 bar and 200 bar for the PMMA, PTHF and 1,2-PB. See Table SI-2 for the parameters used for each polymer.

Table SI-2: Parameters a and ρ_0 leading to the best fit of the PVT data calculated for $X_A = 1{,}000$. Polymers for which surface pressure- mean area per repeat unit isotherms are studied in this work are shown in bold.

Polymer	molecular mass of a repeat unit (g/mol)	a (10^{-20}J)	ρ_0 (10^{27}m^{-3})	$\sqrt{\theta}$ ($10^{-3} g/cm^3$)
LDPE<br>*low density* - Polyethylene	28.05	−1.839	20.948	1.79
i-PP<br>*iso*-Polypropylene	42.08	-2.090	13.436	2.27
PEO<br>Polyethyleneoxide	44.05	-1.923	17.258	1.67
**PB 87% 1-2 (13% 1,4)**<br>**1,2-Polybutadiene**	**54.09**	**-1.815**	**11.360**	**0.65**
1,4-PB<br>1,4-Polybutadiene	54.09	-1.796	11.491	1.25
PIB<br>Polyisobutylene	56.11	-2.025	11.096	0.42
**PTHF**<br>**Polytetrahydrofuran**	**72.11**	**-1.970**	**9.182**	**2.98**
**PDMS ($X_A = 230$)**<br>**Polydimethylsiloxane**	**74.11**	**-1.570**	**9.289**	**1.94**
PVAc<br>Polyvinylacetate	86.09	-1.919	9.412	4.15
PECH<br>Polyepichlorhydrin	92.52	-2.103	9.918	2.62
**PMMA**<br>**Polymethylmethacrylate**	**100.12**	**-2.490**	**7.867**	**2.32**
PS<br>Polystyrene	104.1	-2.430	6.700	2.41

PEMA<br>Polyethylmethacrylate	114.14	-2.372	6.407	4.69
PPO<br>Poly(phenylene oxide)	120.16	-2.414	6.039	8.31
PcHMA<br>Polycyclohexylmethacrylte	168.23	-3.012	4.192	2.02

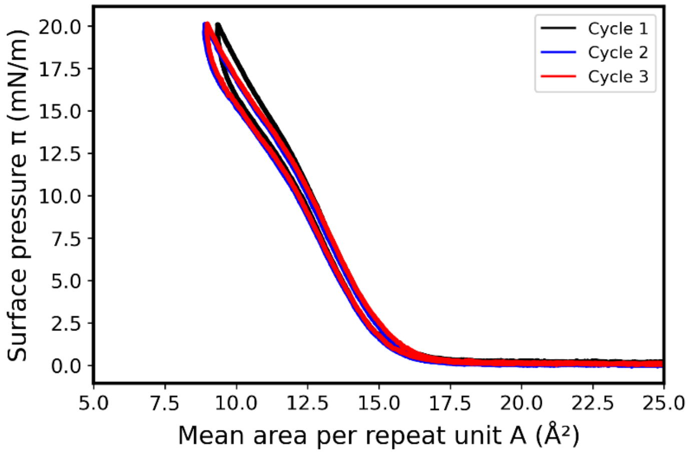

Figure SI-4: Three successive experimental compression-expansion hysteresis cycles at 20 mN/m of the PMMA monolayer.
